# A new practical approach using TeamSTEPPS strategies and tools: – an educational design

**DOI:** 10.1186/s12909-023-04803-2

**Published:** 2024-01-04

**Authors:** Mehrnoosh Khoshnoodifar, Navaz Emadi, Leili Mosalanejad, Sara Maghsoodzadeh, Nasrin Shokrpour

**Affiliations:** 1 E Learning Department, Virtual School of Medical Education and Management. Shahid, Beheshti University of Medical Sciences, Tehran, Iran, Islamic Republic of; 2 E-Learning in Medical Education, Department of E-Learning in Medical Education, School of Medicine, Tehran University of Medical Sciences, Tehran, Iran, Islamic Republic of; 3 Curriculum Planning, Medical Education Department, Jahrom University of Medical Sciences, Main Campus, Motahari Street, Jahrom, 7414813946 Iran, Islamic Republic of; 4 General Psychology, Research Centre for Neuromodulation and Pain, Shiraz, Iran, Islamic Republic of; 5 Teaching English As a Foreign Language, Department of English Language, School of Paramedical Sciences, Clinical Education Research Center, Shiraz University of Medical Sciences, Shiraz, Iran, Islamic Republic of

**Keywords:** Instructional design, Virtual reality, Interprofessional, TeamSTEPPS, Active learning

## Abstract

**Background:**

Teamwork has played a critical role in ensuring patients’ safety and preventing human errors in surgery. With advancements in educational technologies, including virtual reality, it is necessary to develop new teaching methods for interpersonal teamwork based on local needs assessments in countries with indigenous cultures. This study aimed to design and develop a new method of teaching teamwork in cesarean section surgery using virtual reality; we further evaluated the effects of this method on healthcare professionals’ knowledge and attitudes about teamwork.

**Methods:**

This study was designed using the ADDIE instructional design model. The TeamSTEPPS Learning Benchmarks questionnaire was used to assess the educational needs of 85 participants who were members of the cesarean section surgery team. A specialized panel analyzed the extracted needs, and the scenario was compiled during the design stage. Finally, four virtual reality contents were created using 360-video H.265 format, which were prepared from specified scenarios in the development of the educational program. The TeamSTEPPS Learning Benchmarks questionnaire was used to measure knowledge, and the T-TAQ was used to measure the participants’ attitudes.

**Results:**

Six micro- skills were identified as training needs, including briefing, debriefing, cross-monitoring, I'M SAFE checklist, call-out and check-back, and two-challenge rule. Intervention results showed that the virtual reality content improved teamwork competencies in an interprofessional team performing cesarean section surgery. A significant increase was observed in the mean score of knowledge and attitude after the intervention.

**Conclusion:**

Through addressing the need for teamwork training, utilizing the TeamSTEPPS strategy, and incorporating new educational technologies like virtual reality, the collaboration among surgical team members can be enhanced.

## Background

Effective collaboration and mutual understanding between treatment teams have significantly increased the patients’ satisfaction, reduced the costs, improved the patient care, and reduced the patients’ mortality [1]. On the other hand, evidence shows that poor teamwork increases the likelihood of medical errors [2]. As a crucial and sensitive aspect of healthcare provision, the operating room accounts for a considerable proportion of medical negligence cases, with 50% of such cases related to this setting [3]. Many of these negligence cases are associated with non-technical skills among surgical teams [4, 5]. Cesarean section is one of the most common surgical procedures in the operating room, with many pregnant women opting for this method. Therefore, it is the most prevalent surgical procedure among women [6].

The surgical team in charge of performing cesarean section surgery consists of multiple healthcare professionals, including gynecologists, anesthesiologists, operating room nurses, anesthetists, and midwifery experts [7]. Due to the complexity of the surgical procedure and stressful environmental conditions in which it takes place, effective communication and teamwork among team members are essential for achieving optimal outcomes [8]. However, achieving optimal teamwork does not occur haphazardly and necessitates training and planning [8]. Various programs have been developed and implemented to address this issue to improve teamwork and communication among surgical teams [9]. One of the most common methods for strengthening teamwork in medical environments involves teamwork training. Evidence has shown that such training can improve communication among operating room staff, reducing surgical complications and patient mortality [9–13]. One of most effective Strategy for training surgical team members is TeamSTEPPS (Team Strategies and Tools to Enhance Performance and Patient safety) [14]. This program is a systematic approach created by the Agency for Healthcare Research and Quality (AHRQ) to implement teamwork and improve quality, safety, and efficiency in the healthcare system [14–16]. TeamSTEPPS is composed of four learnable skills: Communication, Leadership, Situation Monitoring, and Mutual Support. These skills are essential for achieving knowledge, attitude, and team performance [17]. There are several methods for implementing the TeamSTEPPS teamwork program [18]. McEwan et al. classify teamwork training programs into four categories, including training team members in a classroom setting, utilizing more interactive methods such as workshops, using simulation training, and training on-site [19]. With the advancement of technology, more effective teaching tools have been created to enhance learning using simulation. One of these technologies is virtual reality, which reduces the gap between real and virtual environments, thereby turning the surrounding world into a virtual environment [20]. Virtual reality can be effective in improving team performance in three ways. First, it gives users a real understanding of the environment and ongoing activities from all angles. Second, it provides users with multiple sensory feedback, such as visual and auditory cues, creating a feeling of presence in the real environment. Finally, it strengthens situational awareness and monitoring, which is one of the main competency in training teamwork [21–24]. However, before implementing a new educational approach and specific strategy for teaching teamwork, it is crucial to have an Instructional design. Teamwork results from a combination of knowledge, skills, and attitudes of different team members, emphasizing the importance of identifying the necessary knowledge, skills, and attitudes of team members to design effective teamwork training programs [25]. It is important to note that introducing new technologies, such as virtual reality, into the educational environment does not automatically transform the teaching and learning system in medicine [26]. In fact, virtual reality technology cannot be used in medical education without paying attention to Instructional design components [27]. Using this technology in medical education does not necessarily guarantee the quality of students' learning [28]. Focusing too much on technology and neglecting Instructional design are among the basic problems in designing medical education courses [29]. One solution is to use Instructional design and its models [25, 30, 31]. Therefore, the present study aimed to design a virtual reality content for teaching the TeamSTEPPS strategy for the members of a cesarean section surgery team as an interprofessional skill. More specifically, we aimed to develop a new method of teaching teamwork in cesarean section surgery using virtual reality and further evaluate the effects of this method on healthcare professionals’ knowledge and attitudes about teamwork.

## Methods

### Participants and setting

The participants in this study included all healthcare professionals who worked in surgical teams for cesarean surgery in hospitals. The inclusion criterion was the individuals involved in the cesarean section surgery of a hospital as a team member since the composition of the team is almost fixed in special surgeries like cesarean section. The exclusion criterion was unwillingness to cooperate. The personnel who were interested in traditional education, not technology-based education was excluded from the study after initial interviews. The census method was used to ask the whole research population about their needs. All individuals involved in the cesarean surgery team in the teaching hospitals of Iranshahr University of Medical Sciences, Eastern Iran, were enrolled in this study. The cesarean section team includes a gynecological surgeon, an anesthesiologist, an operating room nurse, an anesthesia nurse, and a midwifery nurse. This study was conducted in 2 hospitals affiliated with Iranshahr University of Medical Sciences.

All operating room staff from 2 hospitals were included in the needs assessment study as a census sample of 85 subjects, out of whom 76 participated in the first part of the study. with a 10% attrition. Then, they were allocated to two 2 teams. One of the team was randomly selected as the experiment group and the other one was the control group.

### Educational intervention

#### The ADDIE stages

Educational design is a method used to analyze the functional problems of learners in a structured way, identify the causes of the problems, and then solve them with a solution with the least unwanted side effects. Functional problems are sometimes rooted in the lack of knowledge and often due to environmental problems. This study used the ADDIE educational method to design an appropriate educational intervention, consisting of five stages: analysis, design, development, implementation, and evaluation. Each stage is explained in detail in the following section [32–34].

### Analysis

The tool used for collecting the needs assessment was the standard test of TeamSTEPPS Learning Benchmarks, which was provided electronically to the members of the cesarean surgery team to determine their educational needs (Table 1).

**Table 1:** Needs assessment of the participants

Sex (%)	Team(%)		Dimensions	Subdomain	Number of questions
Female 82.8% Male 17.2%	operating room nurse	42.1%	**Team Structure**	-	**4**
	anesthesia nurse	19.7%	**Leadership**	Brief-(3)	**4**
				Debrief-(1)	
	Midwifery nurse	15.7%	**Situation Monitoring**	Cross monitoring-(2)	**5**
				STEP-(2)	
				I'M SAFE Checklist-(1)	
	obstetricians and gynecologists	14.7%	**Communication**	Call out & Check-Back-(2)	**3**
				SBAR-(1)	
	anesthetists	7.8%	**Mutual Support**	**Two-Challenge Rule-(3)**	**7**
				**CUS-(1)**	
				**Feedback-(1)**	
				**Assertive Statement-(1)**	
				**DESC Script-(1)**	
**Total questions** 23					

### Design

Demographic information (age, gender, marital status, education, profession, employment status, and work experience) was also collected using seven questions at the beginning of the questionnaire to determine the characteristics of the participants. All operating room staff from 2 hospitals were included in the needs assessment study as a census sample of 85 subjects, out of whom 76 participated in the first part of the study.

### Development

After determination of the priorities of teamwork training using the TeamSTEPPS strategy, an 8-member expert panel meeting was held, including a gynecological surgeon, an anesthesiologist, an operating room expert, a neuroscientist, a midwifery expert, a medical education specialist, an operating room training supervisor, and a facilitator. Based on the strategies and tools of the TeamSTEPPS program, a scenario was developed to train the extracted needs in three stages before, during, and after surgery. The scenario was executed for an experienced cesarean section team, including an anesthesiologist, a gynecological surgeon, an operating room nurse, an anesthetist, and a midwifery nurse. Then, the trained team executed the scenario in a simulated environment. At the same time, the recording was done with a 360-degree camera. Finally, virtual reality content was produced based on the recorded scenario.

### Implementation

This step is a quantitative pre-test and post-test intervention to determine the effectiveness of virtual reality content.. The intervention was carried out in the operation room of Iran hospital under the supervision of Iranshahr University of Medical Sciences (Southeast of Iran). The target group of this study was all the people involved in the surgical teams in cesarean surgery of this hospital. Therefore, based on the composition of the cesarean surgery team, 35 people involved in cesarean surgery, from the combination of 6 gynecological surgeons, 5 anesthesiologists, 6 midwifery nurse, 12 operating room nurse, and 6 anesthesia nurse were selected for this study. The entry criteria was people who were involved in caesarean section surgery of each hospital and were part of the team, because the composition of the team was almost fixed in special surgery like caesarean section. The exit criterion was considered unwillingness to cooperate. Before the intervention, the results of the educational needs assessment using the TeamSTEPPS learning criterion questionnaire were considered as the knowledge score before the intervention, and the average attitude score before the intervention was measured with the standard T-TAQ questionnaire. The intervention in this study consisted of 4 sessions, each lasting of one hour, each before the start of the work shift in the operating room. For the intervention, all team members simultaneously installed virtual reality glasses on their heads and observed their roles in the team with virtual reality (10 min for each session). Then, a discussion for better implementation was held under the supervision of an instructor. Techniques of the TeamSTEPPS strategy in three stages (preoperative, intraoperative (before skin incision), and postoperative) were presented to the cesarean team during the surgeries (at least two operations) of the day. During two sessions, it was first examined in terms of covering educational goals and then in terms of the quality of the produced content. In order to evaluate the educational effectiveness, one week after the intervention, we evaluated their knowledge (TeamSTEPPS criterion) and attitude (T-TAQ) were evaluated similarly to the pre-test.

### Evaluation

The evaluation includes evaluating the validity of the content and evaluating its educational effectiveness. The produced content was given to a team of 10 people including two medical education specialists and 8 people from the selected and experienced cesarean surgery team from Iranshahr University of Medical Sciences, who were at an average level of ability from the researcher's point of view, to check the content and technical validity. During the two sessions, it was first examined in terms of covering educational goals and then in terms of the quality of the produced content. In order to evaluate the educational effectiveness of the content, one week after the intervention of knowledge evaluation (TeamSTEPPS learning criterion), attitude (T-TAQ) was conducted similar to the pre-test.

### Data collection

The TeamSTEPPS Learning Benchmarks test was used as the data collection tool in this study. This electronic test was made available to the surgical team members to determine their training needs. Each hospital had a nominal group, and each participant gave a score to express the importance of each item and determine the spectrum of sub-items. This test measures teamwork skills in four areas: leadership skills, situation monitoring, mutual support, and communication. The test consists of two sets of multiple-choice questions, with 15 questions about teamwork readiness and eight about teamwork knowledge. Each dimension includes six items scored using a 5-point Likert scale (1 = strongly disagree, 2 = disagree, 3 = neutral, 4 = agree, and 5 = strongly agree with the statement). This test is an appropriate tool created by the Agency for Healthcare Research and Quality (AHRQ) to evaluate teamwork readiness and knowledge [15].

The mean score of attitudes before the intervention was measured with the standard Teamwork Attitudes Questionnaire (T-TAQ). The instrument assesses the attitudes of learners towards the areas covered in the transactional strategy, specifically the dimensions of team structure, leadership, situation monitoring, and mutual support. The results identified the surgical team's training needs, and, accordingly, the educational content and implementation methods were designed. The total reliability of the tool was estimated to be 0.80 using Cronbach's alpha, and the ICC was found to be 0.8. Hence, this tool is valid and reliable for measuring attitudes towards teamwork in an Iranian context. Therefore, researchers and practitioners in Iran can use this tool for assessing attitudes toward teamwork in their settings [35, 36].

### Statistical analysis

SPSS version 22 was used for data analysis. Mean (SD) or median (first quartile and third quartile) was used to describe quantitative variables according to the conditions, and frequency (percentage) was used for qualitative variables.

A paired t-test or its non-parametric equivalent, the Wilcoxon signed-rank test, was utilized to compare the pre- and post-intervention results. The dependent t-test was employed when the data exhibited normal distribution, whereas the Wilcoxon signed-rank test was used for non-normally distributed data or small sample sizes. These tests were conducted to ascertain whether there was a noteworthy enhancement in knowledge and attitudes concerning teamwork following the intervention.

## Results

In the educational needs assessment, 76 out of 85 subjects (89%) from the research population participated in the needs assessment as a sample group. Nine participants (10%) refused to cooperate in this research due to their busy schedules. Therefore, the educational needs assessment was done on 76 people.

Among the 76 participants, 50% were in the age range of 20 to 30 years, 82.8% were female, 53.9% were married, and 60% had a bachelor's degree. In terms of the type of profession in the operating room, 42.1% were operating room experts, 19.7% were anesthesiologists, 15.7% were midwifery experts, 14.4% were obstetricians and gynecologists, and 7.8% were anesthetists. Also, The most common work experience was less than 5 years, which included 44.7% of the subjects.

### Results of TeamSTEPPS Learning Benchmarks Questionnaire (AHRQ)

**Fig. 1:**
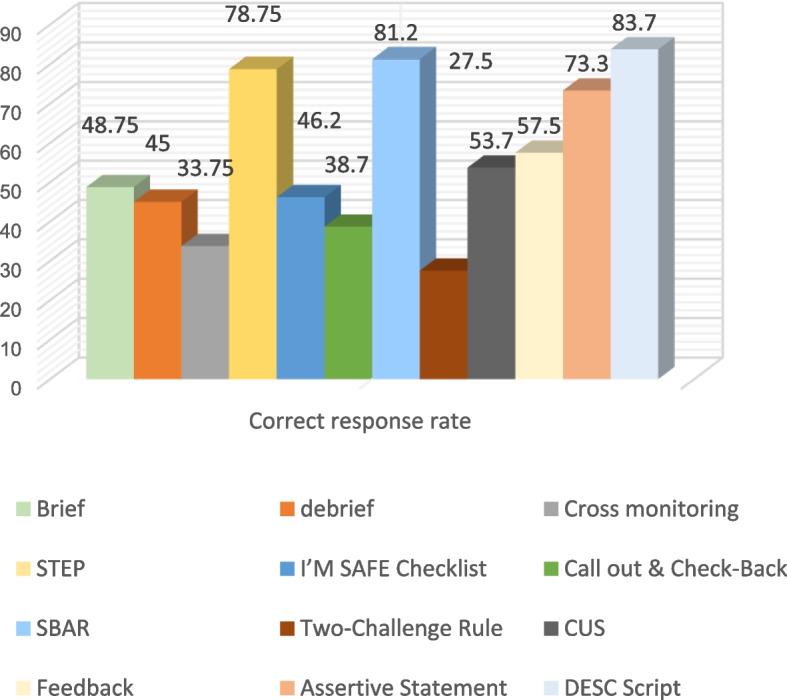
Educational needs of teamwork based on the questionnaire criteria

According to the scores obtained in each section of the questionnaire, each subcategory must receive at least 50% of the total score. Based on the percentage of the scores obtained, the content related to those subcategories with less than 50% was selected for training purposes to address the knowledge gaps among the members. In fact, these contents address the members' educational needs and knowledge gaps. Figure 1 illustrates the relationship between the chosen content and the identified knowledge gaps.

### Effect of the program

The study involved 35 cesarean section team participants, including 6 gynecological surgeons, 5 anesthesiologists, 6 Midwifery nurse, 12 operating room nurse, and 6 anesthesia nurse. The mean age of the participants was 31.88 years. Of the 35 participants, 12 (35%) were male, and 23 (65%) were female.

The study evaluated the impact of virtual reality training on the participants’ knowledge and attitude about teamwork. The results showed a significant increase in the mean score of knowledge and attitude about teamwork after the intervention. Before the intervention, the mean score of knowledge about teamwork in the virtual reality group was 11.8. However, after the intervention, it increased to 18.3 (*P* < 0.0001).

**Fig. 2:**
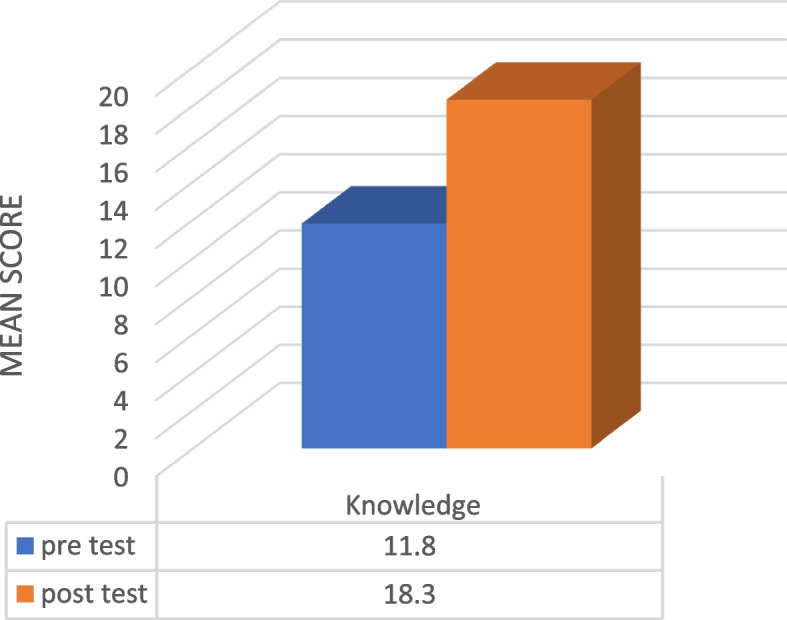
The mean score of teamwork knowledge before and after the intervention

**Fig. 3:**
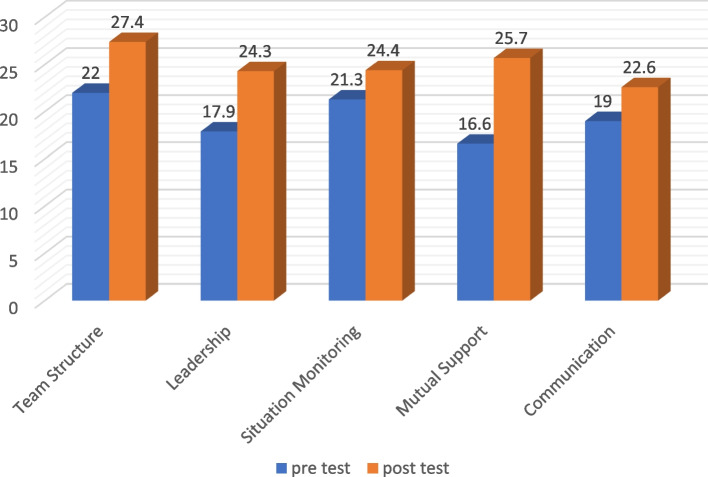
The mean score of teamwork attitude before and after the intervention

The study also evaluated the impact of the intervention on team’s attitude in five areas, including team structure, leadership, situation monitoring, mutual support, and communication skills. The results showed a significant improvement in the mean score of team’s attitude in all five areas after the intervention (Table 2). The details of the results are presented in Figs. 2 and 3.

**Table 2:** Knowledge and attitude of people before and after intervention

Variables		Virtual reality	Mean ± Sd
Knowledge		Before intervention	11.8 ± 3.54
		After intervention	18.3 ± 3.94
		*P*-Value^*^	0.001
Attitude	Structure	Before intervention	22. ± 5.332
		After intervention	27.4 ± 3.767
		*P*-Value^*^	0.001
	Leadership	Before intervention	17.9 ± 5.308
		After intervention	24.3 ± 3.343
		*P*-Value^*^	0.001
	Monitoring	Before intervention	21.3 ± 5.336
		After intervention	24.4 ± 5.210
		*P*-Value^*^	0.023
	Support	Before intervention	16.6 ± 5.865
		After intervention	25.7 ± 4.741
		*P*-Value^*^	0.001
	Communication	Before intervention	19 ± 5.283
		After intervention	22.6 ± 5.801
		*P*-Value^*^	0.014

^*^P-Value < 0.05

## Discussion

In the first part, we reviewed similar articles in the field of study design and implementation model and compared and evaluated the importance of these models in the implementation of the program. The present study evaluated the training needs of the cesarean surgery team members, using the TeamSTEPPS learning criteria questionnaire. Six micro- skills were identified as training needs, including briefing, debriefing, cross-monitoring, I'M SAFE checklist, call-out and check-back, and two-challenge rule. In light of the extracted needs, the virtual reality content was designed to address the identified educational needs.

The present study identified communication skills as one of the primary factors in establishing interaction among operating room teams. However, the scores for communication skills were below the quorum, indicating an educational need. This finding is in the same line with that of a cross-sectional study by Kalantari et al., which reported low scores for communication skills in surgical teams. The study recommended using educational strategies to improve teamwork and communication in operating rooms [37].

The present study proposed a call-out and check-back technique for communication skills training in response to the identified need. The technique was delivered as a scenario, and virtual reality content was developed for it.

The current study identified similar needs for teamwork training as the main obstacles to teamwork in Mir Moulai's study. These needs include monitoring the team's position and providing mutual support in work teams, which can lead to interprofessional and individual conflicts when not properly addressed. These findings suggest that the needs for teamwork training are predictable, but they are highly dependent on the working environment and team structures that determine teachable and learning competency among the team members.

One of the strengths of the present study is the use of a standard teamwork evaluation checklist derived from the TeamSTEPPS, a teamwork training strategy developed by the Health Care Quality and Research Institute [17]. Previous studies used educational design models for producing virtual reality content, such as the ADDIE model, or created models for designing virtual reality content [38–40].

The present study used the ADDIE educational design model to design and produce virtual reality content. This approach is similar to the components identified in another study, which found five main components for designing medical education based on virtual reality: analysis, design, preparation and production, support and implementation, and evaluation [27].

In the next stage of the study, we compared the impact of the intervention on users' knowledge and attitude with other studies and their results.

The present study found that the intervention with virtual reality films increased the mean score of team’s knowledge and attitude, and this effect was statistically significant. Several studies have demonstrated the effectiveness of virtual reality in training, including the studies which are in the same line with the present study [24, 41–44].

A study investigated virtual reality technology in training medical groups for laparoscopic surgery training. The results showed that virtual reality reduced the time spent on training medical groups and played an important role in improving different medical groups [44]. The results of this study were consistent with those of the present study, which used virtual reality to teach the TeamSTEPPS strategy scenario.

Tedson's study used 360-degree videos of implementing a safe surgery checklist to teach teamwork and Patient Safety in Surgical Education. The positive experience of the learners was recorded after using this video, which increased their knowledge, attitude (bilateral support), and team behavior. Similarly, the present study showed 360-degree videos of TeamSTEPPS teamwork training strategy scenarios for the cesarean surgery team [42].

Similarly, another study explored the perceived experience of medical students about communication competence through virtual reality scenarios on Inter-professional care (IP). Students reported the value of learning through this 360 VR approach and emphasized the importance of prioritizing communication within the interprofessional care [41].

Another study presented virtual reality videos used in palliative and oncology medical education. In this study, an effective and acceptable teaching environment was assessed. Students viewed the radiation therapy experience from the patient's perspective. They also used a VR headset and gave a 27-min presentation about the patient's nausea and vomiting experience. A large number of students have introduced this method to their university.

In addition, it is considered to be better than the lecture method and they consider it an innovation in teaching and learning method [43]. The study also demonstrated the potential of virtual reality in meeting these needs and improving the teamwork of the interprofessional team.

Few articles have discussed the disadvantages of technologies such as augmented reality. Some studies have highlighted the negative impact of augmented reality technology on education, including technical complexity, difficulties, multi-tasking, and teacher resistance. These findings underscore the need for expert implementation and performance of technology-based educational interventions. Adopting new technologies in education requires careful consideration of potential risks and benefits [45].

The results of a meta- analysis suggest that the implementation of Augmented Reality (AR) should be approached with caution and undergo a thorough evaluation. The results indicate that AR applications increase the students' academic success in the learning process compared to traditional methods. It was concluded that AR applications did not show a significant difference in academic achievement during the learning process. For example, the "field-level" variable of the study did not show significant differences from traditional methods [46].

Overall, the present study highlights the importance of identifying and addressing the training needs of the cesarean surgery team members. Further research is needed to explore the long-term benefits of virtual reality training for the performance and outcomes of the cesarean section surgery team. Virtual reality technology in medical education can provide learners with a safe and realistic environment to practice and improve their skills. The positive results of studies suggest that virtual reality can be an effective tool for teaching teamwork and communication skills and improving patient safety in surgical training. The importance of collaboration among individuals in developing and implementing programs, especially in team-based studies, is highlighted as a significant and noteworthy factor in the present study.

### Limitations and suggestions for the future

One of the limitations of this study is that it was performed in a group selected from caesarean section. More research is needed to examine the needs of groups in other interprofessional teams. Further research is also needed on the impact of the intervention on group indicators.

However, it is important to localize them to specific culture in each region and clinical environment to ensure the effectiveness of these programs. This can help ensure that the training is relevant and tailored to the specific needs of the healthcare professionals in that setting.

It is essential to consider the obstacles and disadvantages of any technology and cultural approach. In this regard, it is crucial to stay up-to-date with the latest knowledge and address the challenges and problems to create a suitable platform for implementation. Moreover, the real impact and effectiveness of technology deployment can only be assessed by its actual performance. This plays a crucial role in the efficient functioning of technology and interventions.

There is a need to investigate the effectiveness of TeamSTEPPS in various healthcare settings, including hospitals, outpatient clinics, and long-term care facilities. Also, it is suggested that further studies should be conducted on its effects on team performance and its indicators in different team environments to investigate its impact in the cultural context.

## Conclusion

In today's complex healthcare environment, there is a need to strengthen teamwork among healthcare professionals. Specialization and changes in care delivery environments can create distance between different professions, negatively impacting patient care and outcomes.

Overall, the present study's findings suggest that virtual reality technology can effectively improve teamwork and communication skills among healthcare professionals. The TeamSTEPPS teamwork training strategy can also provide a systematic approach to developing these skills. However, to ensure the effectiveness of these programs, we need to localize them to specific culture of each region and clinical environment.

## Data Availability

It is important to note that the datasets used and analyzed during the current study are available from the corresponding author upon reasonable request. This is an important aspect of research transparency, allowing other researchers to verify and replicate the study findings. By making the datasets available, other researchers can conduct further analyses or use the data for other purposes, contributing to advancing knowledge in the field. However, it is important to ensure that the confidentiality and privacy of the study participants are protected when sharing the datasets. Overall, sharing the datasets is an important step in promoting research transparency and scientific integrity, and it can help ensure that the study findings are robust and reliable.

## Abbreviations

TeamSTEPPS: Team Strategies and Tools to Enhance Performance and Patient Safety
T-TAQ: TeamSTEPPS Teamwork Attitudes Questionnaire
ADDIE: Analysis, Design, Development, Implementation, and Evaluation
AHRQ: Institute of Healthcare Research and Quality
